# A Miniaturized Multi-Parameter Synchronous Observation System for In Situ Ocean Turbulence Measurement

**DOI:** 10.3390/s26092654

**Published:** 2026-04-24

**Authors:** Weihong Ouyang, Zengxing Zhang, Junmin Jing

**Affiliations:** Pen-Tung Sah Institute of Micro-Nano Science and Technology, Xiamen University, Xiamen 361102, China

**Keywords:** ocean turbulence, miniaturized system, MEMS sensor, multi-parameter, synchronous measurement

## Abstract

**Highlights:**

**What are the main findings?**

- A miniaturized (70 × 7.7 cm) in-situ ocean turbulence observation system is developed, integrating MEMS 2D turbulence, CTD and attitude sensing modules to realize five-parameter synchronous acquisition.
- High-precision and spatiotemporally synchronized measurements of shear, temperature, salinity, depth and orientation are achieved with 1 kHz high-frequency sampling for turbulence signals.
- Field performance of the system is validated at 1800 m depth in the northern South China Sea through CTD comparison, dual-probe consistency analysis and Nasmyth spectrum fitting.

**What are the implications of the main findings?**

- Intensified shear effects are found to induce enhanced turbulent activity near the thermocline at about 125 m depth.
- This system provides a high-spatiotemporal-synchronization and reliable solution for studying multiscale ocean turbulence and associated dynamic processes.

**Abstract:**

A miniaturized (70 × 7.7 cm) multi-parameter synchronous observation system was developed for in situ ocean turbulence measurement, integrating micro-electromechanical system (MEMS)-based two-dimensional (2D) turbulence, pressure, temperature, conductivity, and attitude sensors. Field tests conducted at a depth of 1800 m in the northern South China Sea validated the system’s accuracy through comparisons with standard CTD (Conductivity, Temperature, and Depth) sensors, dual-probe consistency analysis, and Nasmyth spectrum fitting. The system precisely captured thermoclines, internal waves, and turbulent shear fluctuations at a depth of approximately 125 m, revealing enhanced turbulence near the thermocline due to intensified shear effects. With high spatiotemporal synchronization and reliability, the system provides an effective solution for studying multiscale ocean turbulence and associated dynamic processes.

## 1. Introduction

Ocean turbulence is a fundamental process in physical oceanography, governing the vertical and lateral transport of momentum, heat, salt, and dissolved substances. It plays a critical role in shaping global ocean circulation, climate systems, and biogeochemical cycles [1,2]. In stratified water columns, turbulent eddies spanning small to intermediate scales dominate the dissipation of kinetic energy and modulate thermohaline gradients, thereby controlling the magnitude and efficiency of ocean mixing processes [3]. Furthermore, turbulent fluxes contribute to the breakdown of density interfaces, influence layer stability, and regulate the distribution of nutrients and oxygen in marine ecosystems [4]. Stratification itself exerts strong control over turbulent development. While stable density gradients generally suppress vertical mixing, they can also become regions of intense turbulence when subjected to external forcing mechanisms, such as internal wave breaking or shear-driven instabilities occurring near pycnocline boundaries. Key parameters, including temperature, salinity, and hydrostatic pressure, are intrinsically linked through the equation of state of seawater, making simultaneous, high-resolution measurements essential for accurately characterizing turbulent mixing processes [5]. For example, reliable computation of salinity requires co-located measurements of conductivity, temperature, and pressure. Therefore, spatiotemporally synchronized multi-parameter observations are indispensable for elucidating the mechanisms underlying turbulent mixing and stratification dynamics.

Current methodologies for observing ocean turbulence primarily rely on microstructure profilers equipped with fast-response thermal or shear sensors, as well as remote sensing instruments such as acoustic Doppler velocimeters. Among these approaches, shear probes based on airfoil technology have been widely employed to resolve microscale velocity gradients and estimate turbulent kinetic energy dissipation rates [6]. Despite their high sensitivity and proven performance, such instruments suffer from several limitations, including high manufacturing and operational costs, restricted directional resolution (typically limited to one-dimensional measurements), and insufficient flexibility for scalable or distributed deployments. To address these challenges, the main objective of this study is to develop a novel, miniaturized observation platform that integrates a micro-electromechanical system (MEMS)-based two-dimensional turbulence sensor with standard oceanographic sensors for pressure, temperature, and conductivity, as well as a nine-axis inertial measurement unit [7].

The resulting five-parameter system achieves real-time, synchronous data acquisition of turbulent shear vectors, temperature, depth, salinity, and instrument attitude within a compact, battery-powered profiler. With a total length of 70 cm and a diameter of 7.7 cm, the device is specifically designed to facilitate ease of deployment and scalability in a wide range of oceanographic surveys. Key innovations include: (i) a bionic ciliary MEMS shear sensor capable of resolving orthogonal components of turbulent flow; (ii) an ultra-fast thin-film platinum resistance thermometer incorporating dynamic compensation using the fireworks algorithm [8]; (iii) a seven-ring platinum electrode conductivity cell with board-level temperature compensation; and (iv) a robust STM32-based data acquisition and storage architecture enabling high-frequency sampling, up to 1 kHz for shear measurements, together with a time-stamped file management. Field trials conducted at a depth of 1800 m in the Shenhu area validated the system’s performance through inter-sensor consistency, benchmarking against commercial CTD [9] (Conductivity, Temperature, and Depth) data, and spectral analysis. The results demonstrate the system’s capability to capture fine-scale oceanographic phenomena and provide new insights into the interactions between thermoclines and turbulent shear [10].

## 2. Materials and Methods

### 2.1. System Design

**Figure 1:**
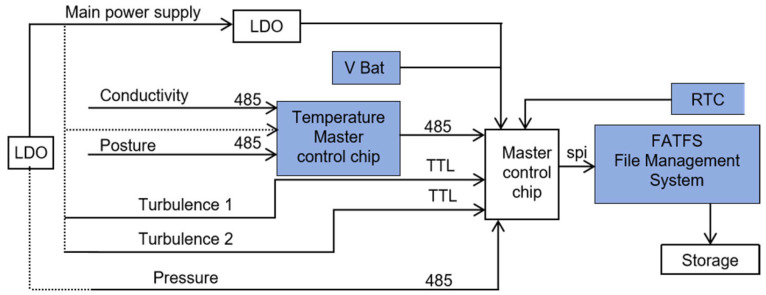
Block diagram of the integrated hardware logic system. The solid lines represent data communication paths and main power routing, while the dotted lines indicate the power supply distribution to individual sensors.

The overall architecture of the instrument comprises three primary functional units: a power supply module, a sensor data acquisition module, and a data storage module (Figure 1).

The power supply module consists of a main power source and a backup battery (Vbat). During normal operation, the main power source drives the entire system. In the event of shutdown or power loss, the backup battery maintains power to the real-time clock (RTC), ensuring accurate timestamping of stored data files. This dual-power design guarantees temporal continuity and reliable synchronization across successive data segments.

The sensor data acquisition module incorporates five submodules: turbulence, temperature, pressure, conductivity, and attitude sensors. Each sensor communicates through its native protocol (UART, RS-485, or I^2^C), with adaptive interfacing managed by the central controller. Low-frequency parameters, including temperature, pressure, conductivity, and attitude, are sampled at 0.5 Hz, whereas high-frequency turbulence signals are continuously acquired at 1 kHz [11].

The data storage module utilizes an SPI (Serial Peripheral Interface, a synchronous serial communication bus that enables high-speed data transfer between the STM32 microcontroller (STMicroelectronics, Geneva, Switzerland) and the SD card, supporting efficient time-series data logging in this instrument) interface to communicate with an SD card, enabling high-speed writing of time-series data. An FATFS file system implemented on an STM32 microcontroller organizes all data into sequentially named files, each corresponding to a continuous observational segment. Each data frame includes millisecond-precision timestamps in its header, ensuring accurate temporal alignment across multiple sensor streams. To prevent data loss during high-rate sampling, a ring-buffer mechanism temporarily buffers incoming turbulence data before writing them to storage.

Upon activation, the main controller initializes all sensor modules through programmable electronic switches, allowing flexible configuration according to specific mission requirements. After instrument recovery, the recorded data can be retrieved directly via a waterproof connector on the tail cap, which supports both wired communication and external power input.

This system design satisfies the dual requirements of asynchronous low-frequency sampling and continuous high-frequency recording, while simultaneously simplifying post-processing workflows through a unified and consistent data structure.

**Figure 2:**
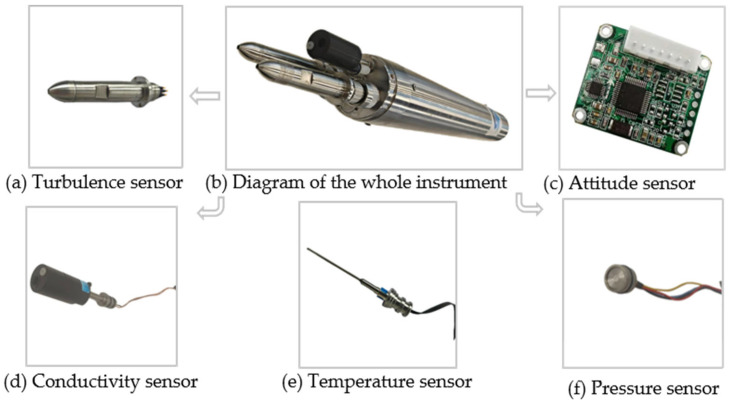
Schematic illustration of the complete instrument and sensor layout. The arrows point to the sub-figures, which detail the individual sensor modules equipped on the instrument.

The structural configuration of the integrated instrument is illustrated in Figure 2. The device adopts a three-section design, with an overall length of 70 cm and a diameter of 7.7 cm. The front sensor base houses two turbulence sensor probes, one temperature sensor probe, one conductivity sensor probe, and one pressure sensor probe. Sensor circuits, power supplies, and attitude sensors are enclosed in a cylindrical, pressure-resistant housing with a wall thickness of 5.5 mm. Verified through three independent pressure test cycles, the hull ensures stable operation at water depths of up to 5000 m. A watertight connector on the tail cover enables power control and direct data retrieval through a cable interface. Compared to conventional microstructure profilers (e.g., the Rockland VMP-250, which typically exceeds 130 cm in length and 14 kg in weight), the proposed system’s dimensions and lightweight design (~4.5 kg) represent a significant reduction in form factor. This miniaturization, achieved through the integration of compact MEMS sensors, facilitates hand-deployment and improves the scalability for distributed oceanographic surveys.

### 2.2. Sensor Selection and Configuration

Turbulence Sensors: Custom-developed MEMS-based 2D turbulence sensors were employed, drawing inspiration from the mechanoreceptive function of fish lateral line cilia [12]. Each sensor features a cross-beam structure with piezoresistive elements arranged in Wheatstone bridge configurations along two orthogonal axes. Fluid-induced deflections generate strain within the cantilever beams, producing differential voltage outputs proportional to the local shear components in the x- and y-directions. This configuration enables decomposition of omnidirectional turbulent flows into orthogonal vector components, enabling true 2D vector sensing. Each sensor achieves a spatial resolution of 3 mm and a response time of 1 ms, facilitating the detection of microscale turbulent structures [13].

Temperature Sensor: An ultra-high-precision thin-film platinum resistance temperature sensor was selected for its linearity, repeatability, and rapid thermal response [14,15]. Temperature is derived from resistance variations using a precision current excitation circuit. The sensor operates over a temperature range of −5 °C to 35 °C, with an accuracy of ±0.003 °C and a response time of 68 ms, enabling the resolution of subtle thermal microstructures. Dynamic error correction is applied using a fireworks algorithm-based compensation model to mitigate transient thermal lag during rapid vertical profiling.

Conductivity Sensor: A custom quartz-tube seven-platinum-ring conductivity cell was developed based on the dual-electrode alternating current (AC) measurement principle [16]. Alternating current is applied across the outer electrodes to minimize polarization effects, while the inner electrode rings measure the resulting voltage drop. In combination with a PT5000 reference resistor, a board-level temperature compensation algorithm corrects thermal drift in real time. The sensor covers a conductivity range of 0–90 mS/cm, with an accuracy of ±0.003 mS/cm and a response time under 50 ms, making it suitable for capturing sharp halocline transitions.

Pressure Sensor: A piezoresistive thin-film pressure transducer was used for depth measurement [17]. Internal silicon strain gauges form a Wheatstone bridge, two arms of which consist of pressure-sensitive resistors. Applied hydrostatic pressure induces resistance imbalance, generating a differential voltage output proportional to the applied pressure. The signal is amplified and linearized onboard. The sensor provides a full-scale measurement range of 0–100 MPa with a precision of 0.01%, corresponding to a depth resolution better than 1 m throughout the full ocean water column.

Attitude Sensor: A nine-axis inertial measurement unit (SEC290, Beiwei Sensing Technology Co., Ltd., Wuxi, China) was integrated to monitor pitch, roll, and yaw angles in real time. Following factory calibration and field validation, the sensor achieves an angular resolution of 0.01° and a repeatability of 0.1°, ensuring accurate tracking of platform orientation during free fall [18].

### 2.3. Sensor Layout

The strategic arrangement of sensors within the observation system plays a critical role in ensuring data fidelity and minimizing interference among measurement modules. In this design, particular attention was devoted to the spatial configuration of the turbulence, temperature, conductivity, pressure, and attitude sensors to preserve hydrodynamic integrity and optimize signal quality [19]. Two MEMS-based 2D turbulence sensor probes were symmetrically mounted on opposite sides of the instrument’s front section, enhancing measurement redundancy and enabling robust cross-validation of shear data. This bilateral configuration mitigates directional bias and improves the reliability of vector turbulence detection under varying flow orientations. Meanwhile, the temperature, conductivity, and pressure sensors were co-located on the same horizontal plane at the leading edge of the instrument to ensure that all scalar parameters were sampled from nearly identical water masses, thereby maintaining spatiotemporal coherence. By positioning the turbulence sensors slightly upstream and elevated relative to the CTD-type sensors [20], the influence of flow distortion caused by wake effects from adjacent probes is significantly reduced. Furthermore, this spatial separation minimizes the potential impact of mechanical vibration or electromagnetic interference on sensitive thermal and conductive measurements. The attitude sensor, enclosed within the central pressure-resistant housing, benefits from a stable internal environment that effectively shields it from external pressure fluctuations and biofouling. This carefully engineered layout not only supports high-fidelity microstructure observations but also conforms to established best practices in oceanographic instrumentation, where probe placement directly affects the quality of derived turbulence parameters such as dissipation rates and diffusivity estimates.

### 2.4. Selection of Main Control Chip

**Figure 3:**
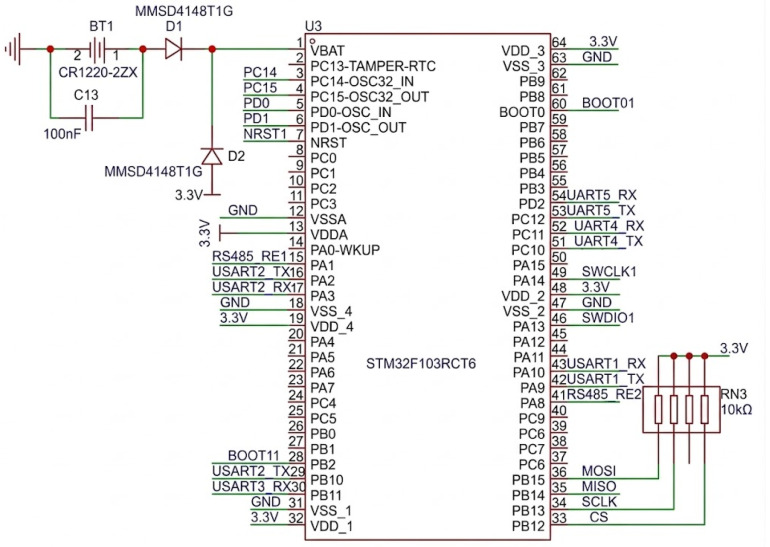
Main control circuit. The numbers indicate the pin assignments of the microcontroller, and the green lines represent electrical connections.

The STM32F103RCT6 microcontroller (STMicroelectronics, Geneva, Switzerland) (Figure 3) serves as the central control unit of the multi-parameter observation platform, coordinating real-time data acquisition, communication protocol management, timestamp generation, and high-speed storage across heterogeneous sensor modules. Given the stringent requirement for concurrent sampling at markedly different frequencies, ranging from 0.5 Hz for low-frequency environmental parameters to 1 kHz for high-frequency turbulent shear signals, the selection of a microcontroller with adequate processing capability, memory resources, and peripheral support was critical. The STM32F103RCT6, based on the ARM Cortex-M3 architecture (Arm Ltd., Cambridge, UK), offers a favorable balance between computational performance and low power consumption, making it particularly suitable for long-duration autonomous underwater deployments. Its five USART interfaces allow independent serial communication with multiple sensors, including dual turbulence probes, temperature and conductivity acquisition boards, and the nine-axis attitude sensor, without the need for additional multiplexing hardware. The integration of an external RS-485 transceiver provides noise-resistant differential communication for long-distance transmission of pressure and temperature data from remote sensor heads, which is particularly advantageous in electrically noisy underwater environments. Moreover, interfacing the SD card storage module via an SPI bus supports sustained write speeds sufficient to accommodate the high-volume data stream generated by the 1 kHz turbulence channels. Combined with the FATFS file system implemented on-chip, this architecture ensures reliable, sequential data logging with minimal latency and negligible packet loss [21,22]. A reserved UART channel further enhances system extensibility, allowing for future integration of auxiliary sensors or real-time telemetry through acoustic modems. Overall, the selection of this microcontroller reflects a pragmatic and forward-looking approach to embedded system design for oceanographic instrumentation, in which reliability, scalability, and precise timing are paramount [23].

## 3. Results and Discussion

### 3.1. Experimental Setup and Deployment Procedure

Field validation was conducted on 6 February 2025, in the Shenhu area (111°28.25′ E, 18°03.64′ N), a mid-slope region of the northern South China Sea with a total water depth of approximately 1970 m. Two prototype turbulence profilers and a reference CTD48M (Sea & Sun Technology, Trappenkamp, Germany) were deployed simultaneously in a vertical profiling mode. The deployment occurred between 08:17 and 09:28 CST, with a target profiling depth of approximately 1800 m. All instruments descended freely at a controlled rate of approximately 0.5 m/s using adjustable ballast, powered by internal batteries and communicating in real time via a 50 m tether. Post-recovery procedures included powering down the data acquisition module, rinsing the instruments with fresh water, and extracting raw data from the onboard SD cards. The CTD data were downloaded separately for subsequent comparative analysis.

### 3.2. Operational Workflow

The operational workflow of the miniaturized multi-parameter profiler was carefully designed to ensure procedural consistency, data integrity, and instrument safety throughout the entire deployment cycle. Prior to immersion, a comprehensive pre-deployment checklist was completed, including visual inspection of seals, verification of O-ring integrity, torque checks on fasteners, and confirmation of watertight connector functionality. Powering on the system initiated internal diagnostic routines, during which communication links between the main controller and each sensor submodule were verified using handshake protocols. Real-time telemetry via a 50 m tether enabled surface personnel to monitor sensor outputs and overall system health before release, ensuring readiness for profiling operations. Upon deployment, the instrument commenced free-fall descent through the water column, continuously recording synchronized measurements while maintaining precise temporal alignment through millisecond-resolution timestamps embedded in each data frame. After completion of the observational dive, the instrument was recovered using a flotation-assisted retrieval system. Immediately upon surfacing, power to the signal acquisition module was manually disconnected to preserve data integrity and prevent potential corruption during post-recovery handling. The instrument was then thoroughly rinsed with fresh water to remove salt deposits and prevent corrosion, particularly around exposed sensor interfaces and electrical contacts. Finally, data extraction was performed by removing the SD card and transferring raw binary files to a secure workstation for decoding, time synchronization, and preprocessing. This standardized workflow minimizes human error, protects instrument longevity, and ensures dataset traceability and reproducibility, key requirements for scientific rigor in field oceanography.

### 3.3. Descent Velocity Analysis

**Figure 4:**
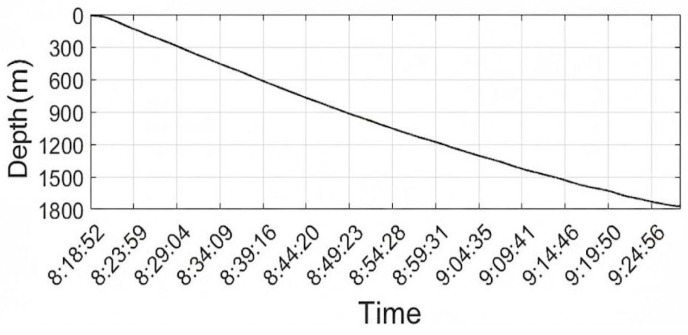
Depth profile of the turbulence profiler during free descent.

**Figure 5:**
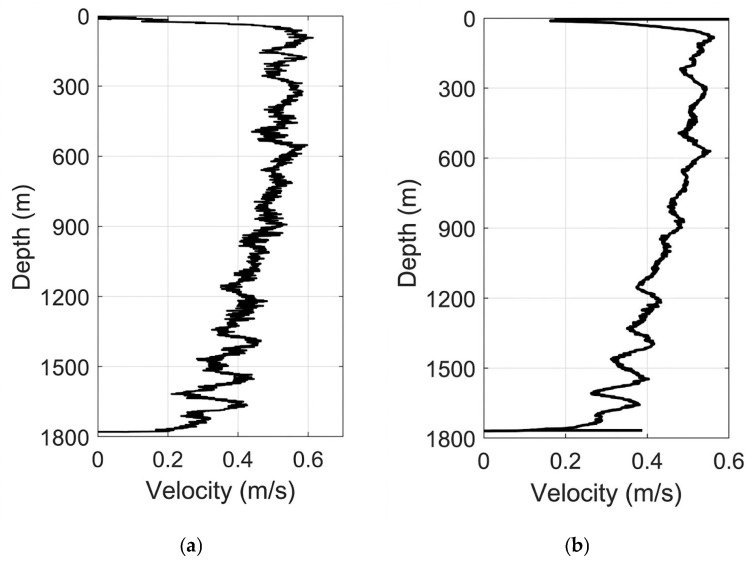
Descent speed profiles: (**a**) self-developed profiler; (**b**) co-deployed standard CTD sensor.

Accurate characterization of the profiler’s descent velocity is essential for converting temporal sensor records into depth-resolved profiles and for interpreting turbulence statistics within the correct spatial framework. During vertical free-fall observations, depth data were continuously logged using the onboard piezoresistive pressure sensor, sampled at 0.5 Hz and converted to depth using the UNESCO equation of state for seawater (Figure 4). Differentiation of the depth profile yielded the instantaneous descent velocity, revealing distinct kinematic regimes through the water column. Between depths of 100 m and 600 m, the instrument maintained a highly stable sinking rate with an average velocity of approximately 0.53 m/s, indicating a dynamically balanced configuration with minimal oscillation or rotation. This stable phase corresponds to the upper thermocline and mixed layer transition zone, where ambient density gradients are relatively weak. Below 600 m, the descent velocity gradually decreased to an average of about 0.4 m/s, likely attributable to increased fluid drag associated with lower temperatures and higher viscosity in deeper, denser water masses. Despite this gradual deceleration, the descent trajectory remained smooth and non-oscillatory (with a minor fluctuation standard deviation of approximately 0.015–0.02 m/s), with no abrupt spikes or stalls indicative of entanglement or instability. The overall mean descent velocity of approximately 0.45 m/s falls within the optimal range for microstructure profilers, balancing spatial resolution against mission duration. Crucially, comparison with the concurrently measured descent velocity from the reference CTD sensor (Figure 5) demonstrated strong agreement in both trend and magnitude, confirming the accuracy of the pressure-derived depth estimates and validating the profiler’s hydrostatic performance. These results demonstrate that the instrument maintains a predictable and stable kinematic profile throughout its descent, which is a prerequisite for acquiring high-quality turbulence measurements.

### 3.4. Temperature and Conductivity Comparison Experiment

**Figure 6:**
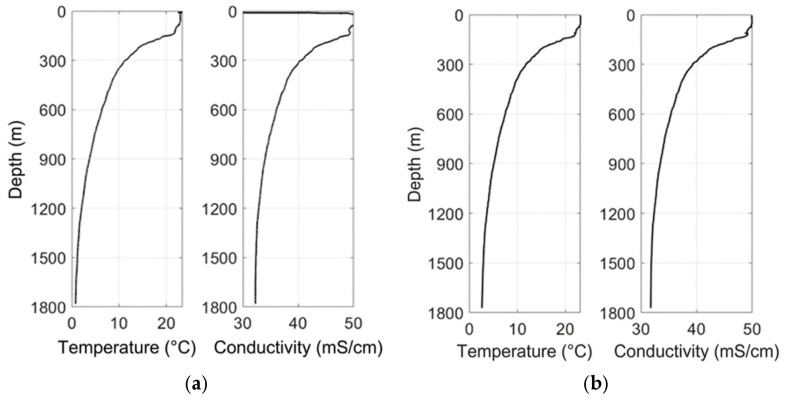
Temperature and conductivity profiles: (**a**) self-developed profiler; (**b**) reference CTD sensor.

To evaluate the accuracy and consistency of the custom-developed temperature and conductivity sensors, a side-by-side comparison was performed against a commercially calibrated CTD48M instrument during simultaneous vertical profiling (Figure 6). Across the full depth range of 0–1800 m, both systems captured highly coherent thermal and haline structures. The most prominent feature, a sharp thermocline observed near 127 m in the CTD record, was consistently reproduced by the self-developed profiler at approximately 125 m, with nearly identical temperature gradients, characterized by a decrease from approximately 23.5 °C at the surface to about 5.7 °C below the pycnocline. Similarly, the conductivity profiles exhibited a monotonic decrease from approximately 50.1 mS/cm at the surface to 32.2 mS/cm at depth, closely matching the CTD measurements, which decreased from 50.0 to 31.7 mS/cm over the same interval. These minor discrepancies fall well within the specified measurement uncertainties of both instruments (±0.003 °C for temperature and ±0.003 mS/cm for conductivity), indicating negligible systematic bias. The tight correlation between the datasets underscores the effectiveness of the board-level temperature compensation applied to the platinum-ring conductivity sensor and validates the dynamic calibration methodology based on the fireworks algorithm used for the thin-film platinum resistance thermometer. Notably, the absence of phase lags or anomalous spikes in the profiles indicates reliable temporal synchronization and minimal thermal lag, even during rapid transitions across sharp gradients. Collectively, these results demonstrate that the custom-developed sensors perform comparably to established commercial systems, reinforcing confidence in their suitability for quantitative investigations of fine-scale oceanographic structures, such as double-diffusive staircases or thermohaline intrusions.

### 3.5. Shear Data Analysis

**Figure 7:**
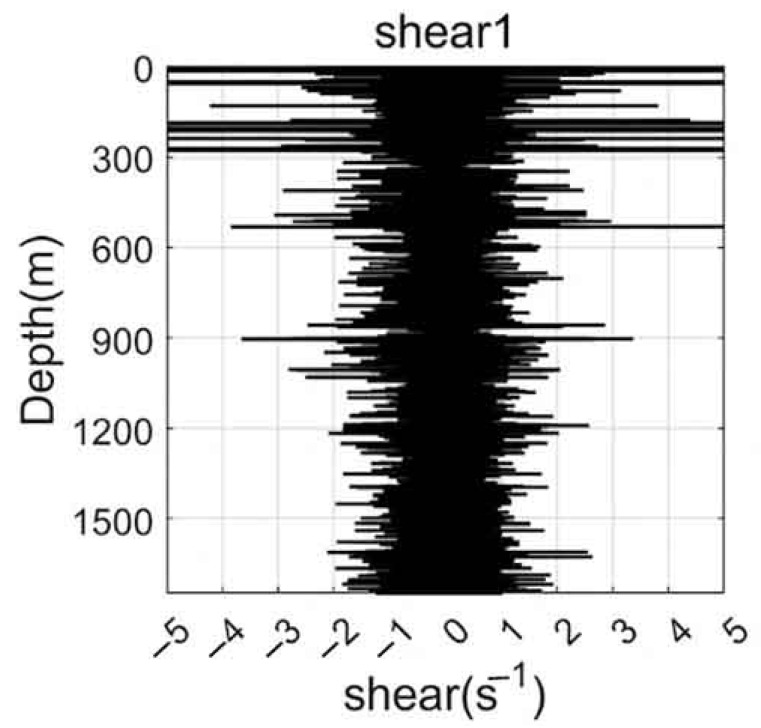
Shear fluctuation profile recorded by Shear Probe 1 of the self-developed turbulence profiler during observation.

**Figure 8:**
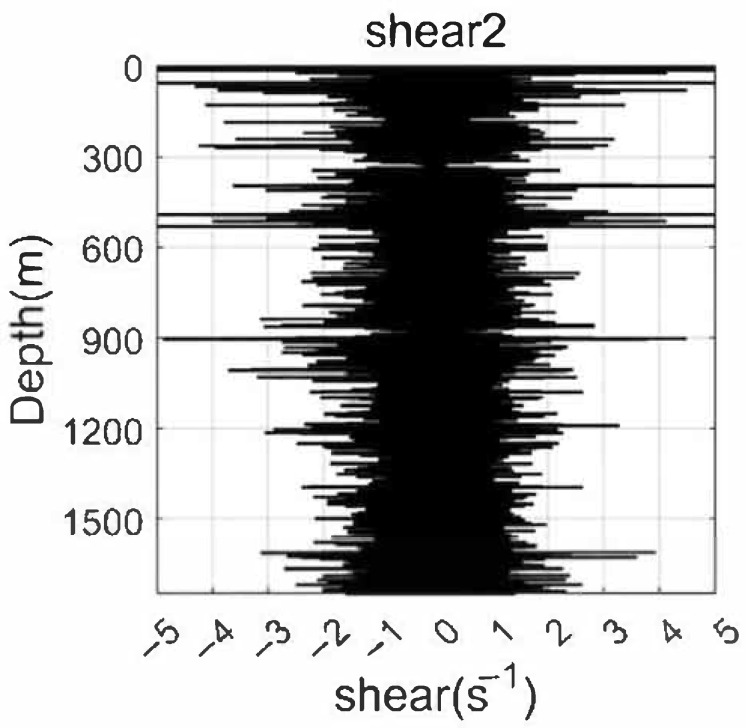
Shear fluctuation profile recorded by Shear Probe 2 of the self-developed turbulence profiler during observation.

Shear data collected by the dual MEMS-based 2D turbulence sensors provide valuable insights into the small-scale dynamics of oceanic flows, as shown in Figure 7 and Figure 8. The vertical profiles of turbulent shear, recorded at a sampling rate of 1 kHz, reveal characteristic patterns associated with different physical processes. Near the surface, pronounced fluctuation peaks are observed immediately after water entry, primarily resulting from wave-induced accelerations and splash-related disturbances. These transient effects decay rapidly with increasing depth, giving way to background turbulence levels that are more representative of ambient flow conditions. Of particular interest is the emergence of significant shear fluctuations near the thermocline depth (approximately 125–150 m), where enhanced velocity gradients coincide with strong stratification. These localized bursts of shear variability suggest the presence of shear-driven instabilities, potentially associated with nonlinear internal waves or Kelvin–Helmholtz billows, which are well-recognized precursors to turbulent mixing. At greater depths (exceeding 600 m), the shear signals become progressively weaker, reflecting the stabilizing influence of reduced current velocities and energy input. Nevertheless, intermittent episodes of elevated shear persist, possibly indicating sporadic mixing events driven by tidal forcing or geostrophic adjustment processes. A key advantage of the dual-probe configuration is its capacity for intra-instrument validation. The two independent shear channels exhibit nearly identical fluctuation patterns in terms of amplitude, spectral content, and temporal occurrence, demonstrating strong mutual consistency and high sensor reliability. This built-in redundancy not only increases confidence in the observed shear features but also enables future application of advanced signal processing techniques, such as coherence analysis or spatial gradient estimation.

### 3.6. Shear Spectrum Fitting Analysis

**Figure 9:**
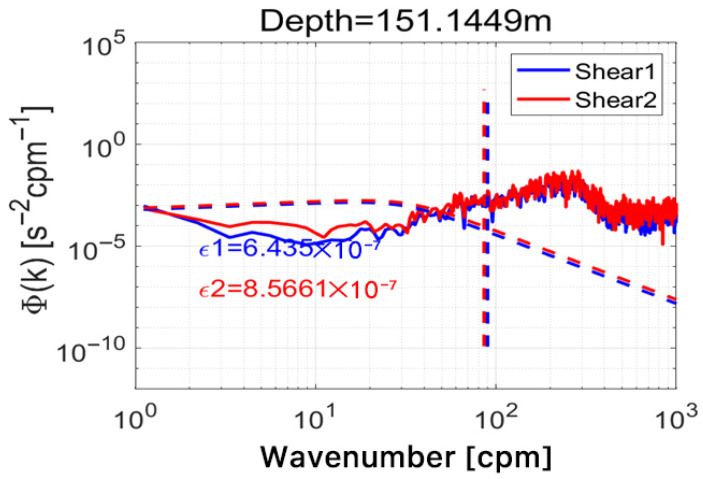
Shear wavenumber spectra at 150 m depth: comparison between dual MEMS probes and the canonical Nasmyth spectrum. The oblique dashed curves represent the theoretical Nasmyth spectra fitted based on the estimated dissipation rates, while the vertical dashed lines indicate the corresponding cutoff wavenumbers.

Spectral analysis provides a robust framework for validating turbulent shear measurements by comparing observed wavenumber spectra with established theoretical models. In this study, as shown in Figure 9, shear data collected near 150 m depth, within the primary thermocline region, were subjected to spectral decomposition and fitted to the canonical Nasmyth spectrum, which characterizes the universal form of shear variance in fully developed ocean turbulence. The wavenumber spectra derived from both turbulence probes exhibit a clear roll-off at high wavenumbers, following the expected −1 slope within the inertial subrange, and show close agreement with the Nasmyth model across overlapping spatial scales. This strong correspondence indicates that the measured shear fluctuations conform to established turbulence scaling laws and are not dominated by instrumental noise or spectral aliasing. The quality of the spectral fit further confirms that the MEMS-based sensors possess adequate sensitivity, bandwidth, and dynamic range to resolve relevant turbulent eddies down to the Kolmogorov microscale. Additionally, the close agreement between spectra from the two independent probes reinforces the conclusion that the system reliably captures genuine hydrodynamic signals rather than spurious electronic or mechanical artifacts. Deviations from the theoretical spectrum at very low or very high frequencies remain minimal, suggesting effective signal conditioning and appropriate anti-aliasing measures within the data acquisition chain. Collectively, these results affirm the scientific validity of the shear dataset and support its use in deriving quantitative turbulence parameters, such as the turbulent kinetic energy dissipation rate (ε) and vertical diffusivity (Kρ), which are essential for parameterizing mixing processes in ocean circulation models.

## 4. Conclusions

A miniaturized, five-parameter ocean turbulence observation system capable of simultaneous, high-resolution measurements of 2D turbulent shear, temperature, depth, salinity, and instrument attitude was developed and successfully field tested. The compact form factor (70 cm × 7.7 cm) and modular sensor integration enable repeated, cost-effective deployments in deep-ocean environments. Field experiments conducted at a depth of 1800 m in the Shenhu area demonstrated stable platform performance, with an average descent velocity of 0.45 m/s. Validation through comparison with a commercial CTD system, internal cross-consistency checks between dual shear probes, and spectral fitting to the Nasmyth model collectively confirm the accuracy and reliability of the measured parameters. Notably, pronounced shear fluctuations were observed near the thermocline (approximately 125 m), highlighting the role of sharp density gradients in amplifying hydrodynamic instabilities and enhancing localized turbulent mixing. These observations underscore the importance of co-located, multi-parameter measurements for understanding the coupling between stratification and turbulence in the ocean interior. Future work will focus on extending the system endurance for long-term moored deployments, further improving energy efficiency and deploying sensor arrays to achieve spatially resolved turbulence mapping. The proposed system represents a significant step toward affordable, scalable, and high-fidelity ocean turbulence monitoring, with broad applications in physical oceanography, climate research, and marine ecosystem studies.

## Data Availability

The raw data supporting the conclusions of this article will be made available by the authors on request.
